# Individual- and Institutional-level Concerns of Health Care Workers in Canada During the COVID-19 Pandemic: A Qualitative Analysis

**DOI:** 10.1001/jamanetworkopen.2021.18425

**Published:** 2021-07-27

**Authors:** Suze G. Berkhout, Kathleen A. Sheehan, Susan E. Abbey

**Affiliations:** 1 University Health Network, Toronto, Ontario, Canada; 2 Temerty Faculty of Medicine, Department of Psychiatry, University of Toronto, Toronto, Ontario, Canada; 3 University of Toronto Institute for the History and Philosophy of Science and Technology, Toronto, Ontario, Canada

## Abstract

**Importance:**

Mental health and coping difficulties among health care workers (HCWs) have been reported during pandemics and particularly during the COVID-19 pandemic.

**Objective:**

To examine sources of distress and concern for HCWs in Canada during the COVID-19 pandemic.

**Design, Setting, and Participants:**

In this qualitative study, a critical discourse analysis was performed of questions posed by HCWs to hospital senior leadership between March 16, 2020, and December 1, 2020, through an online employee forum as part of a larger mixed-methods evaluation of a stepped-care mental health support program for HCWs at 1 of Canada’s largest health care institutions. Questions could be submitted online anonymously in advance of the virtual forums on COVID-19 by any of the University Health Network’s 21 555 employees, and staff members were able to anonymously endorse questions by *upvoting*, indicating that an already posed question was of interest.

**Main Outcomes and Measures:**

Themes, text structure, and rhetorical devices used within the questions were analyzed, taking into consideration their larger institutional and societal context.

**Results:**

Unique individual views of the forums ranged from 2062 to 7213 during the study period. Major individual-level concerns related to risks of contamination and challenges coping with increased workloads as a result of the pandemic intersected with institutional-level challenges, such as feeling or being valued within the health care setting and long-standing stratifications between types of HCWs. Concerns were frequently reported in terms of calls for clarity or demands for transparency from the institutional leadership.

**Conclusions and Relevance:**

The findings of this qualitative study suggest that larger institutional-level and structural concerns need to be addressed if HCWs are to be engaged in support and coping programs. Potential service users may be dissuaded from seeing their needs as being met by workplace mental health interventions that solely relate to individual-level concerns.

## Key Points

**Question:**

What have been the range of concerns and the sources of distress among health care workers in Canada as the COVID-19 pandemic has evolved?

**Findings:**

In this qualitative study of a public online COVID-19 forum available for 21 555 employees in a university health network, common concerns relating to the pandemic included risks of contamination, appropriate personal protective equipment, and worker safety. Although these concerns manifested as individual distress, they also intersected with and were reflective of concerns relating to health care institutions’ policies, communication practices, and politics.

**Meaning:**

The findings suggest that a mismatch between institutional sources of concern and individual-level interventions may affect the uptake of mental health supports even as the level of distress remains high.

## Teaser

This qualitative analysis uses an online COVID-19 forum available to employees at a large health care institution to examine sources of distress and concern for health care workers in Canada during the COVID-19 pandemic.

## Introduction

With ongoing social and economic restrictions, stay-at-home orders, and an increasingly burdened health care system, the COVID-19 pandemic has been an affront to the mental well-being of many individuals.^1,2^ Among health care workers (HCWs), adverse psychological outcomes, including anxiety, depression, insomnia, and burnout, have been widely reported.^3,4^ In areas with high COVID-19 exposure and case volume, HCWs are especially likely to experience distress.^5^ Extensive pandemic media coverage and the widespread use of social media may also contribute to perceptions of risk and difficulties coping.^6^ Despite this, the uptake of supports by HCWs has remained limited even when these are offered at no or low cost.^7^ Moreover, to our knowledge, no evidence-based interventions for HCWs during pandemic events were evaluated before the COVID-19 pandemic. A recent Cochrane review^8^ suggests that, in general, resilience-promoting training programs for HCWs have little or no effect on anxiety, well-being, or quality of life and provide only low-certainty evidence for subjective improvements in perceptions of resilience and depression. A consistent mismatch remains between high levels of distress among HCWs and low-quality evidence for how to manage this situation.^7^ During the second and third waves of the COVID-19 pandemic, contending with the range of perpetuating factors that contributed to mental distress among HCWs became a challenge. In this qualitative study, we sought to understand the range of concerns and sources of distress among HCWs at the University Health Network (UHN) in Toronto, Ontario, Canada, as the COVID-19 pandemic evolved and asked how these concerns might intersect with one another in the institutional context.

## Methods

### Overview

In this qualitative study, as 1 component of a mixed-methods evaluation of an internal HCW mental health support program at UHN (the UHN COVID CARES program), we conducted a critical discourse analysis (CDA) of a series of online open forums (virtual forums) hosted by senior hospital leadership. The eMethods in the Supplement gives additional details. The study was approved by the UHN Quality Improvement Review Committee and received a formal waiver from research ethics board review because this study was undertaken for the purposes of program evaluation and quality improvement. A written informed consent process was still performed for all interviews. Data were deidentified at the time of transcription (eTable in the Supplement). This study followed the Standards for Reporting Qualitative Research (SRQR) reporting guideline.^9^

Critical discourse analysis is a qualitative method that enables analysis of associations among language, text, talk, power, and culture; it examines vocabulary, grammar, rhetoric, and text structure within a particular social context.^10^ The method is used to evaluate how text and talk are produced, circulated, distributed, and consumed.^11^ The function of language to shape public perceptions of issues is recognized and the discourse is considered as both constitutive of and constituted by social practices.^12^ Although communicative expression is generated at an individual level, CDA can be used to evaluate this within a larger social, cultural, and political context.

The virtual forums that we examined have been hosted regularly during the COVID-19 pandemic as part of an ongoing communication tool between senior leadership and the 21 555 staff members who work at UHN, a large, multisite health care setting (Table 1). Any staff member can submit a question or concern related to the COVID-19 pandemic through an online platform (slido.com). Staff may submit as many questions to as many forums as they desire, although each question is not read aloud at a virtual forum. Questions submitted in advance of the live-streamed event are reviewed and prioritized by leadership and responded to on camera through a live-streamed video posted to a YouTube channel and maintained for later viewing. One feature of the forums has been *upvoting*: questions can be endorsed anonymously by other staff through online voting. We tracked the upvotes as an indicator of support for a given question because upvotes were used by the leadership team to prioritize questions for response and the process required engagement by staff in advance of a given forum to cast an upvote.

**Table 1.** Characteristics of University Health Network Staff and Employees

Characteristic	Staff and employees, No.
Paid employees	16 978
Administrative or clerical	2253
Allied health or health professions	2922
Information technology	574
Management	923
Medical professionals	474
Ontario Nurses' Association nurse	3360
Professional administrative	1518
Scientific	589
Support services	2636
Technologists or technicians	1729
Unpaid staff	2897
Administration	255
Technologists or technicians	28
Allied health or health professions	85
Scientific	238
Other	2291
Physicians	1680
Total staff and employees^a^	21 555

^a^ Excludes learners and volunteers.

This CDA was embedded within the needs assessment component of the UHN COVID CARES program evaluation. In brief, UHN is a large, publicly funded hospital network that provides tertiary and quaternary care in Toronto, Ontario, Canada. The UHN COVID CARES program was developed as the first wave of the pandemic emerged in Canada (March to April 2020) and consists of (1) UHN CARES, a modified stepped-care model^13^ of individual mental health supports for HCWs; (2) Team CARES, proactive outreach that provides support to clinical areas affected by COVID-19; and (3) the provision of support to those working in support roles (wellness leads, social work staff, and spiritual care services). The qualitative needs assessment consisted of the CDA as well as interviews with a range of hospital staff, managers, and senior leadership team members. An ongoing mixed-methods program evaluation has also been performed. Findings have been reported through leadership channels in addition to contributing to changes within the UHN COVID CARES program.

### Analysis

We examined all questions selected by leadership for the virtual forums from March 16 to December 1, 2020, and tracked the upvotes each question received. Themes were developed through an iterative close reading^14^ of the forum questions and their prioritization. We attended to the word choice, syntax and sentence structure, rhetorical style, and text features (such as bolded or words placed in all capital letters by the writer for emphasis). We paid particular attention to when questions were identified by the author as having been previously submitted to earlier forums. We also situated the forum questions within a broader institutional and societal context; at the start of each forum, the hospital chief executive officer provided a preamble, which we annotated. Comments relating to internal institutional events or external societal issues were cross-referenced with public sources of information, and on 2 occasions, the forums were reported by public media^15,16^ and included in our analysis.

At each iteration of our analysis, annotation was used to refine and link themes. In total, 4 iterations of thematic coding were performed, 2 of which entailed discussions among the authors regarding theme accuracy and completeness. The process concluded once no further modifications to the coding scheme were deemed necessary. Research memos and reflexive notes were tracked by one of us (S.G.B.). Forum questions were triangulated with the UHN COVID CARES needs assessment interviews.

## Results

### Forum Participants

**Figure 1.**
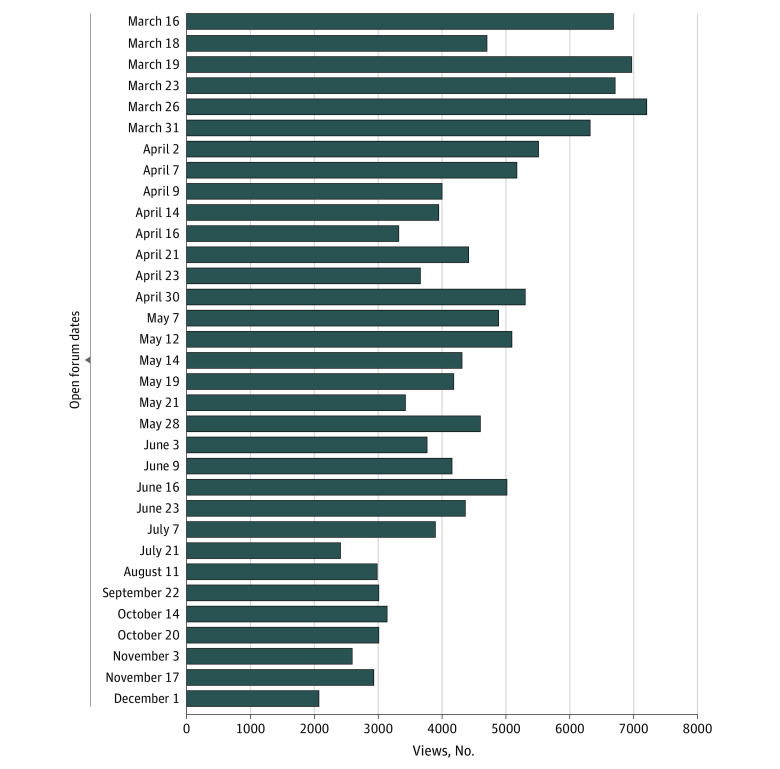
Weekly Virtual Forum Views From March 16 to December 1, 2020

The virtual forum comments revealed that the forums were experienced by staff as an important communication tool and were widely viewed throughout the pandemic (Figure 1). Unique views of the forums (calculated for each forum on the day the data were extracted for analysis) ranged from 2062 to 7213 individuals. This range likely underestimates the total number of individuals viewing at least some part of each forum because these forums were sometimes viewed live by multiple individuals using a single computer (eg, in a nursing station). During the study period, forum questions were submitted and upvoted anonymously; thus, data on the writer of the question were not available unless this information was included in the submission.

### Intersections of Individual-Level Concerns With Structural Issues and Institutional Transparency

**Figure 2.**
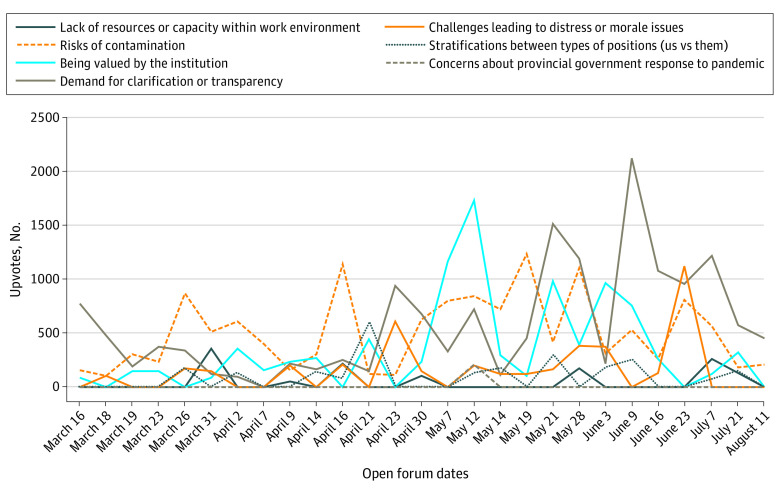
Discourse Analysis Themes and Upvotes by Week During the First Wave of the COVID-19 Pandemic in Toronto, Ontario, From March to August 2020 Upvotes were defined as anonymous endorsement of questions and concerns by other staff through online voting. The first outbreak of COVID-19 occurred at Toronto Western Hospital in April; redeployment, April 23; pandemic pay (financial bonus to frontline, essential workers) removed from certain groups, May; redeployment and long-term care report, from May 14 to June 3; essential care partner policy introduction, June 9; and policies and statements regarding long-term care and Black Lives Matter, from June 23 to August 11.

The tenor of early pandemic-related distress, as demonstrated through questions that used terms such as *worried*, *fear*, *nervous*, and *anxious*, simultaneously revealed fears relating to risks of infection or contamination with SARS-CoV-2 and concerns about a lack of transparency within the institution. The perceived risk to HCW safety was shown by individual concerns about COVID-19 infection that intersected with a broader context of structural and institutional concerns, including supply chain limitations, rapidly changing infection control policies, and increased workflow with a smaller number of HCWs. Requests for information regarding structural and institutional issues frequently engaged language relating to clarity vs perplexity and at times implied that information might have been withheld from staff (Figure 2). As elective and nonemergency procedures were halted and clinics shut down, patients in stable condition were moved to increase bed capacity, and staff redeployment was initiated as the UHN became involved with supporting long-term care facilities in the greater Toronto area, where most COVID-related deaths occurred during the first wave of the pandemic. These scenarios were sources of uncertainty and change that challenged HCWs’ ability to cope and subsequently became central topics in the forums (Table 2). Forum contributors asked explicitly how the institution would support the mental health of HCWs, framing conventional responses such as employee assistance programs, corporate wellness initiatives, and mindfulness practices (which were demonstrated on some virtual forums) as inadequate for meeting HCWs’ needs.

**Table 2.** Qualitative Themes and Selected Virtual Forum Questions

Theme	Quotations (upvotes, No.)
Risk of contamination or safety concerns	“Dr X, you keep deflecting on the risk of pre-symptomatic transmission. But viral loads are shown to be similar between symptomatic and asymptomatic carriers. Now, more recent studies are saying these ‘silent spreaders’ may actually be the MAIN driver of transmission. Why are you minimizing this?” March 18, 2020 (51) “There is recent evidence that shows injury to the placenta in pregnant women with COVID-19. Royal College of Obstetricians strongly recommends pregnant HCW[s] (>28 weeks) avoid direct patient care. With asymptomatic spread and outbreaks on ‘clean floors,’ why is UHN keeping pregnant HCWs at work?” May 28, 2020 (182) “The number of staff with positive tests since January in the corporate intranet is reported at 130. This number was 128 two weeks ago, before the TWH outbreaks and multiple cases that I am aware of at TGH—why are you not accurately reporting staff positivity rate? It is much higher than 1.8%.” October 20, 2020 (69)
Being valued by the institution	“Morale is decreasing in high exposure areas. We feel devalued, unprepared and disposable to the organization. We don’t feel protected and as if our experience and opinions don’t matter. What incentives will you offer to ensure staff retention since you’re not willing to offer pay incentives?” April 14, 2020 (83) “Will UHN please consider paid time off for those staff not receiving pandemic pay [financial bonus to frontline, essential workers] or working from home? I am referring to essential full-time staff who have been coming to work every day and not receiving either of those perks.” May 28, 2020 (174) “Is there anything being done for people working onsite at the peak of the pandemic, but were not eligible for Pandemic Pay? What about people that have no choice but to take the already packed TTC buses and trains? We feel undervalued—as an Admin I received no help whatsoever.” October 14, 2020 (74)
Stratifications between roles or positions (us vs them)	“Staff in different departments are given respite days (PAID days off) due to lower workload in certain areas. Other staff (even in the same dept) do not get respite days because they HAVE TO work the front line. Will these frontline staff get any similar compensation after this pandemic is over?” April 21, 2020 (119) “My direct manager does a great job. But agree with the thought exchange comment: executive team seems out of touch with frontline staff. Why only 1 quest on the [engagement] survey about executive team? Why did the final quest only ask what did UHN do well? AND not about opportunities for improvement?” June 23, 2020 (120) “Some of the comments on this forum during the past few months of COVID have demonstrated a sharper than ever divide between clinical and non-clinical folks. There seems to be a lack of understanding and appreciation for each other. Going forward, how will UHN leadership help to bridge this gap?” July 21, 2020 (151)
Demand for transparency or clarification	“Why are acute care staff training HCWs to support patients in acute care only for our managers to redeploy experienced acute care staff elsewhere? There’s a lack of transparency that is causing confusion, fear, disengagement and safety concerns in acute care. Higher oversight is needed.” April 23, 2020 (210) “I am still struggling to understand the moral argument that UHN will not release the report on TWH outbreaks, just recommendations, because ‘we are afraid of getting sued.’ Is that even valid? It is disrespectful to the staff who were infected to withhold the full report. We want real transparency.” June 3, 2020 (123) “Dr X, thank you for explicitly clarifying the universal masking policy for patients (at all times outside of room). But again, this issue was raised months ago in a previous open forum. Why the delay/hesitation? Why was the change made only now? Did this factor into our recent outbreaks?” November 3, 2020 (101)
Explicit issues of mental distress or morale issues	“What is UHN doing to support the mental health of its staff beyond recommending EAP and a self-meditation tutorial? Many staff will likely require help beyond what can be offered via the EAP program, and our benefits (if we have them) poorly address mental health or provide adequate funds for them.” March 31, 2020 (67) “The mental health of our staff is deteriorating as a result of being redeployed out of their sites, positions, work hours and days. It was okay for a short time but now that the months drag on this is no longer a reasonable ask. Outpatient clients have lost so much therapy (virtual is not the same).” June 16, 2020 (70) “Leadership seems disconnected with frontline staff. To suggest last forum that people have been happy with redeployment on the basis of a couple of anecdotes examples ignores and dismisses the numerous questions & many hundreds of upvotes in all previous forums. Most are not happy with redeployment.” June 23, 2020 (240)
Lack of resources or capacity in clinical environment	“Frontline workload—any initiatives for strategies in place to reduce the number of times staff need to go into isolated rooms and preserve our PPE? Can we tap into the TeleMonitor Team expertise to increase remote observation to help front line nursing workload by decreasing unnecessary exposure?” April 16, 2020 (212) “Thinking about ramping up capacity back to ‘normal’ is making me anxious, are you able to share what the plan is?” April 30, 2020 (103) “Many employees that have been working from home these past 10 weeks with less than full workload. Can they be asked to come and help screen? Can they relieve those of us who are doing their own role + being redeployed? We are burning out while others sit safely. Time to switch places.” May 28, 2020 (170)

Abbreviations: EAP, employee assistance program; HCW, health care worker; PPE, personal protective equipment; TGH, Toronto General Hospital; TTC, Toronto Transit Commission; TWH, Toronto Western Hospital; UHN, University Health Network.

Early concerns about the risks of contamination were also framed in relation to concerns about transparency. Such questions typically had an assertive, interrogative rhetorical style and were often contextualized in relation to competing interpretations of the scientific evidence regarding the pandemic. Subsequent concerns about redeployment, workloads, and patient movement were also framed by explicit demands for transparency from senior hospital leadership or had implicit suggestions that information was being withheld from HCWs in the institution (Table 2). Individual-level concerns about safety, risk, and contamination intersected with institutional-level issues about workflow, staffing levels, and a perception that the pandemic was being used to push through larger, often unpalatable institutional changes that would have otherwise been subject to greater consultation and bidirectional input.

### Concerns During the Second Wave of the Pandemic

**Figure 3.**
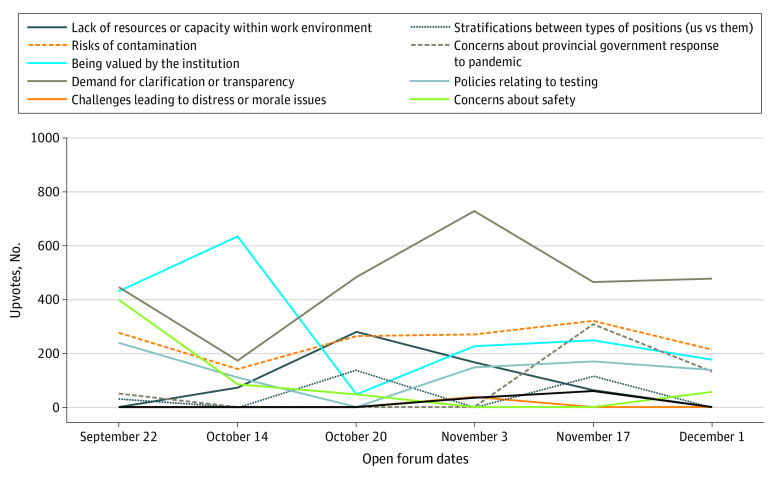
Discourse Analysis Themes and Upvotes by Week During the Second Wave of the COVID-19 Pandemic in Toronto, Ontario, From September to December 2020 Upvotes were defined as anonymous endorsement of questions and concerns by other staff through online voting. Redeployment to long-term care was flagged from September 22 to October 24; vacation carryover policy announced, October; and Public Health Agency of Canada identified aerosols as transmission, November 3.

Intersecting fears about contamination and a perceived lack of transparency led to a broader set of concerns regarding the institutional context and culture, particularly regarding whether and how HCWs were valued within the institutions (Figure 3). As the pandemic evolved, sick leave policies, employee benefits, donations of food and free parking for hospital workers, and which groups of HCWs were eligible for pandemic pay (financial bonus to frontline, essential workers) from the provincial government were addressed in the virtual forums. These topics reflected a broader set of concerns regarding the institutional culture and the valuing of HCWs. Of interest, a practice of providing a “shout-out” to team members or colleagues who were believed to deserve praise had been a practice early in the forums and then ceased to occur for a period. These shout-outs were reintroduced as the second wave progressed.

The forums decreased in frequency through fall 2020 as a second-wave surge of infections affected the region. Demands for clarification or concerns about transparent communication remained consistent, continuing to overlap with themes of risk, safety, and being valued within the institution. Explicit mental health concerns were flagged less frequently during the second wave, although when they were flagged, these concerns were placed in the context of workloads, burnout, and a perception of mismatch between corporate priorities and the demands of care provision placed on frontline workers.

### Social and Structural Context and Expression of Concerns 

Concerns reported by staff were further shaped by events both inside and outside the health care network. Early during the forum implementation, these expressions of concern related to social media representations of the pandemic, hospital disclosures about unit outbreaks, and external reports about infection and mortality rates in long-term care. Through June and July 2020, in line with world events, transparency concerns and how HCWs were valued mapped onto larger societal issues, such as anti-Black racism and the health care system’s role in this racism.^17^ In the second-wave forums beginning in September 2020, an increasingly broad set of themes intersected with HCW concerns about transparency and being valued, including how the hospital system interfaced with governmental policy, particularly as the provincial government was seen to be failing to follow the suggestions of its medical advisers.^18^ Procedures relating to prioritization of COVID-19 vaccination eligibility at the provincial level became prominent, as did comments and criticisms regarding the enforcement of public restrictions, which arose in relation to challenges regarding workloads, fatigue, and burnout.

## Discussion

In this qualitative study, similar to the published literature,^19^ early themes of distress in the UHN’s open forums were reported regarding personal protective equipment and worker safety. These concerns were not only associated with individual anxieties about contamination. High levels of concern involved uncertainties about viral transmission because of overstretched hospital capacities and excessive workloads, disrupted personal protective equipment supply chains, and how these disruptions would be managed in the context of a rollout of widespread social and economic restrictions.^1,20^ Also similar to the published literature^21^ was the evolution of these concerns as the pandemic evolved; sources of distress shifted to include burnout, fatigue, and moral injury associated with the conditions under which care was being provided. During each wave, forum contributors made reference to the limitations of typical or conventional approaches to HCW support, such as employee assistance programs and the promotion of mindfulness practices through wellness initiatives. The perception of these as stopgap measures and inappropriate to the nature of many HCW concerns was echoed in the qualitative interviews conducted as part of the UHN COVID CARES needs assessment and program evaluation.

In both the COVID-19 pandemic and previous pandemics, such as the severe acute respiratory syndrome pandemic, transparency and trust in the institutional setting were identified as key elements to managing fear and uncertainty.^21^ Trust in leadership has also been identified as a component of organizational culture that contributes to mental health outcomes among HCWs.^22^ Of importance, institutional sources of distress for HCWs at the UHN occurred in the context of a hospital-wide move to an incident management system. Sometimes referred to as a command and control approach, the incident management system is a top-down decision model with limited opportunities for feedback.^23^ At the UHN, this model was implemented within a larger context of executive leadership restructuring, which may have enhanced the scale of concerns about transparency and trust. Despite the forums being implemented to increase 2-way communication between leadership and staff, decisions about the forums (eg, changes in format) remained opaque, such that tensions surrounding transparency and trust were understood as important and central themes modeled within the forums’ processes and content.

Topics raised in the virtual forums reflected HCWs’ sense of how they were valued. The sense of being valued likewise intersected with concerns regarding transparency and trust in the institution. As indicated in the virtual forums, distress relating to these intersecting issues may have contributed to burnout and the loss of meaning within one’s work during the pandemic. This finding is similar to that reported in the literature^24^ on the interrelations among moral injury, self-efficacy, and loss of trust in health care institutions. This finding also reflects the ways in which distress and coping can be hindered or facilitated within a given organizational culture, particularly through leadership practices, group behaviors and relationships, and communication and participation.^22^ Notably, the virtual forums not only provided information about the institutional culture through the themes but also contributed to how that culture was shaped.

### Strengths and Limitations

This study has strengths. The analysis provided a novel way of understanding the intersections of individual- and institutional-level challenges experienced by HCWs during the COVID-19 pandemic. This approach allowed the exploration of topics of concern not easily captured by the validated scales and quantitative measures typically used to survey HCWs’ mental health and coping. A strength of the CDA method was the capacity for making conclusions about the relationships between institutional actors and HCWs. The virtual forums were part of the way in which these relationships were structured.

This study also has limitations. Certain groups of employees may not have had access to technology in their work environment, which may have curtailed their ability to engage with the virtual forums. Although the virtual forums were available for viewing and questions could be submitted to slido.com through the external (public-facing) institutional website, this process would require time, computer literacy, and access to technology at home to be used by those without access in their work role. Moreover, many other aspects of the forums (eg, links, summaries of responses, and discussion of the forum content) were often performed by internal all-user emails. During at least some of the study period, not all employees had an institutional email address. The disparities in access to the virtual forums as a communication tool may demonstrate reliability with regard to the themes of stratifications in types of positions (us vs them) and being valued in the institution.

This analysis was also limited by a lack of knowledge about the individuals who posed questions to the forum. Therefore, how this analysis might inform supports for particular populations of HCWs should not be overstated, and generalizations should not be made about the experience of distress by any given type of HCW. The use of methods such as CDA in concert with other approaches (quantitative or qualitative) when applying a CDA to program development would be valuable.

Qualitative research is marked by its inductive analytic practices and by the way in which multiple layers of meaning and interpretation can be explored in a single data set.^25^ The themes described in this article are not the only possible interpretation of the CDA data. Approaching the data set with a different research question would likely yield different themes. However, this study provided an analysis of 1 layer of meaning. At this stage, the analysis only briefly considered the response of leadership to questions. An analysis of the dynamics of question and response would be a valuable subject of further study and would likely yield further findings on the role of leadership and communication in organizational culture.

## Conclusions

Supports for HCWs are typically designed as individual-level interventions; however, in this study, institutional-level stressors also affected HCW distress and coping and intersected with individual-level concerns. The most notable concerns included the ways in which institutional practices conferred or denied value to HCWs and the intersection of fears about worker safety with broader concerns regarding institutional transparency. When staff are using all their waning energy to perform their duties,^26^ reaching out for support may seem like another untenable task, and accessing services is less likely to occur if supports appear as though they fail to align with the source of concern.^7,27^ Maintaining the well-being of a dedicated, effective, and energetic health care workforce may involve addressing institutional challenges in addition to individual symptoms.

## Notes

Supplement. **eMethods.** Supplemental Methods **eTable.** Breakdown of Qualitative Interview Participants within the UHN COVID CARES Program Evaluation
