# Supplementary material for: Individual- and Institutional-level Concerns of Health Care Workers in Canada During the COVID-19 Pandemic: A Qualitative Analysis
Source: JAMA Netw Open. 2021 Jul 27;4(7):e2118425. doi: 10.1001/jamanetworkopen.2021.18425 (PMC8317008; doi:10.1001/jamanetworkopen.2021.18425)
Supplement: Supplement. — eMethods. Supplemental Methods eTable. Breakdown of Qualitative Interview Participants within the UHN COVID CARES Program Evaluation [file jamanetwopen-e2118425-s001.pdf]

Supplementary Online Content

Berkhout SG, Sheehan KA, Abbey SE. Individual- and institutional-level concerns of health care workers in Canada during the COVID-19 pandemic: a qualitative analysis. *JAMA Netw Open*. 2021;4(7):e2118425. doi:10.1001/jamanetworkopen.2021.18425

eMethods. Supplemental Methods
eTable. Breakdown of Qualitative Interview Participants within the UHN COVID CARES Program Evaluation

This supplementary material has been provided by the authors to give readers additional information about their work.

© 2021 Berkhout SG et al. *JAMA Network Open.*

eMethods. Supplemental Methods

Setting

We undertook a critical discourse analysis (CDA) of online open forums ("virtual forums") as one component of evaluating the rapidly developed stepped-care mental health program for HCW at our institution (UHN COVID CARES). Our hospital system includes two general hospitals, a cancer hospital, a rehabilitation institute and a post-secondary educational institute for healthcare professions. As an institution, our setting provided care for SARS patients in 2003, and numerous HCW with SARS experience continue to work within the institution. Throughout the COVID-19 pandemic we have been the provincial resource for extracorporeal membrane oxygenation (ECMO). We have multiple intensive care and medical units dedicated to COVID-19 patients, two emergency departments, a COVID assessment centre for the public, and also operate "hotels" for homeless COVID-19 positive patients. As further context, the health network's leadership structure has undergone significant revision over two years leading into the pandemic, including a new chief executive officer (CEO) and re-structuring of the executive leadership.

The "UHN COVID CARES" program is a multi-faceted support program consisting of a self-referral program with stepped levels of care (Richards 2012), designed to offering increasing "steps" of care as service user needs increase, as well as a range of outreach supports and resources to clinical teams and services within the health network. It was created to complement existing institutional wellness programming and provide a more intensive level of specialist care at the highest steps of the program. Care is provided in collaboration between psychiatrists in the UHN Centre for Mental Health and UHN psychologists whose pre-pandemic roles existed across a range of clinical areas. A core set of individuals from each group were involved in the design of services and self-management resources, in addition to conducting the program evaluation.

Approach

The virtual forums hosted regularly through the pandemic are part of an ongoing communication tool between senior leadership and the 21,555 employees and physicians who work in the institution. The publicly available forums can be viewed in real-time and links to the recordings are disseminated on the public-facing institutional website, the internal "intranet," and via email with noted "key points," editorial clarifications, and time stamps for central issues. The virtual forums were one of the institution-wide communication tools used through the COVID-19 pandemic. The broader communications strategy is largely carried out by email, through the internal institutional intranet, and during area huddles. Virtual forums were frequently referenced in internal institutional emails and are also highlighted on the external-facing (public) institutional website.

Our data set for this CDA includes the publicly available questions posed to leadership between March 16, 2020 and December 1, 2020 that were responded to within the forums, and the upvotes for each question. Questions that made reference to previous forums were analyzed against the particular forum mentioned (most commonly noted when the individual submitting the question felt that their issue had been ignored in a previous week).

© 2021 Berkhout SG et al. *JAMA Network Open.*

The CDA is further situated within our larger program evaluation, which includes qualitative interviews with a range of HCW, managers, physicians, and executive leadership team members, in addition to interviews with UHN COVID CARES service users and service providers. Although interview data is not presented in this analysis, findings from the interviews have been used to triangulate the analysis of the CDA and provide additional context for the questions we have analyzed. See supplemental table 1 for a breakdown of the program evaluation interview participants.

We tracked the thematic content of questions, rhetorical devices and style embedded within the questions, as well as the number of upvotes each question received. We also followed the context of the questions, by examining when editorial comments had been made in the forums, and by examining when questions at a given week made reference to previous questions. Using this approach supported an understanding of both individual and institutional aspects of HCWs' concerns within the pandemic and the relationships between these. Initial themes were generated through close reading of the forum questions by week by SB, which were open coded manually. Developing codes for a given week were cross-referenced with subsequent weeks until a coding tree was established with major themes and subthemes. Themes were refined through team meetings with KS and SA, and the final analysis reached through consensus with all authors. In total, four iterations of coding and revision of themes took place. Although we analyzed and reported only on publicly available information, our own positionalities as physicians with varying seniority and roles in the institution have meant that we have access to institution-wide emails and discussions related to the virtual forums, which may have implicitly shaped our understanding the data. Reliability was enhanced by triangulating open forum themes against individual interviews carried out during the larger program evaluation. This qualitative component of the larger evaluation followed SRQR guidelines for reporting qualitative research (O'Brien et al. 2014).

Ethical Considerations

The project was approved by the UHN Quality Improvement Review Committee and received a formal waiver from research ethics board review (QI ID #20-0069). While REB approval is not required for publicly available information, a perception of exposure may still generate harms for participants (Sugiura et al. 2017). During the period we report on, questions were submitted anonymously, and the discussion we offer focuses on broader themes and exemplars of these, while avoiding any direct quotations that could reflect a work location or unique concern. We also followed the "upvotes" as a marker of overall relevance of a given concern to the HCWs in our institution. These are doubly de-identified and represent anonymous approval of an anonymous question. Expectations of privacy within an online forum are challenging to navigate and in the context of this forum, where transparency was a central theme, the tensions surrounding having forums be both "open" and "safe" (i.e. private) were high.

These tensions arose again in February 2021. The leadership team posted on the YouTube channel that the forums were meant to be "internal" only (though the content remains publicly available) and as of March 2021, individuals posing questions were required to provide their name in order to have their questions posed in the virtual forum. While a rationale was provided, namely that a literature exists that suggests that anonymous questions do not seem to enhance the perception of transparency or trust within an organization, it remains to be seen whether the

© 2021 Berkhout SG et al. *JAMA Network Open.*

move away from accepting anonymous questions to the virtual forums will enhance versus hinder the sense of bidirectional, transparent communication. We have, though, limited our analysis to first and second wave questions which were posed anonymously and we have ceased analyzing any forums from February 2, 2021 onward.

Using The CDA Within Our Larger Program

The CDA we conducted constitutes a novel way of understanding and addressing the intersections of individual and institutional-level challenges. The analysis has informed the development of resources and interventions offered to staff at our health care network through the UHN COVID CARES program, delivered by clinicians within our Centre for Mental Health. This has included providing feedback to clinicians about the CDA itself so that the larger context is understood when individuals seek support, providing occupational health and human resource processes and pathways to clinicians when these avenues were needed for CARES users, expanding team-based supports through drop-ins at unit huddles so that issues and concerns could be debriefed by unit rather than emphasized as individual issues, and feeding forward institutional concerns through management, leadership, and institution communication channels. We were also part of a group advocating for email access for all employees.

eTable 1. Breakdown of Qualitative Interview Participants within the UHN COVID CARES Program Evaluation

Evaluation Component	Participant Role in Institution	Number of Interviews
Needs Assessment	Physician	5
	Nursing (manager)	4
	Nursing (staff)	2
	Other manager/director (environmental services, allied health, occupational health, non-clinical areas)	4
	Executive/Senior Leadership	4
	Other staff	2
UHN CARES Stepped-Care Evaluation	Service User	10
	Service Provider	6

References

O’Brien, BC, Harris, IB, Beckman, TJ, Reed, DA, Cook, DA. “Standards for reporting qualitative research.” *Academic Medicine*. 2014; **89**(9): 1245-1251.

Richards, DA. “Stepped care: A method to deliver increased access to psychological therapies.” *Canadian Journal of Psychiatry* 2012; **75**(4): 210-215.

Sugiura L, Wiles R, and Pope, C. “Ethical challenges in online research: Public/private perceptions.” *Research Ethics* 2017; **13**(3-4): 184-199.

© 2021 Berkhout SG et al. *JAMA Network Open.*
